# Acoustic Performance and Flame Retardancy of Ammonium Polyphosphate/Diethyl Ethylphosphonate Rigid Polyurethane Foams

**DOI:** 10.3390/polym14030420

**Published:** 2022-01-21

**Authors:** Huiping Zhang, Xiongxian LYU, Zijun Huang, Ying Yan

**Affiliations:** Guangdong Provincial Key Lab of Green Chemical Product Technology, School of Chemistry and Chemical Engineering, South China University of Technology, Guangzhou 510641, China; hpzhang@scut.edu.cn (H.Z.); celvxiongxian@mail.scut.edu.cn (X.L.); 201920123080@mail.scut.edu.cn (Z.H.)

**Keywords:** rigid polyurethane foam, acoustic absorption, flame retardant, open-cell structure

## Abstract

Flame-retardant water-blown rigid polyurethane foams (RPUFs) modified by ammonium polyphosphate (APP) and diethyl ethylphosphonate (DEEP) were synthesized by a one-pot free-rising method. We performed scanning electron microscopy (SEM), compression strength tests, acoustic absorption measurements and thermogravimetric analysis, as well as limited oxygen index, vertical burning and cone calorimeter tests to investigate the mechanical properties, acoustic performance and flame retardancy of the foams. SEM confirmed that the open-cell structures of the foams were successfully constructed with the introduction of a cell-opening agent. Upon using 20 php APP, the average acoustic absorption coefficient of the foam reached 0.535 in an acoustic frequency range of 1500–5000 Hz. The results of thermogravimetric analysis demonstrated that the incorporation of APP and DEEP can effectively restrain mass loss of RPUFs during pyrolysis. In particular, the compressive strength of a foam composite containing 5 php APP and 15 php DEEP increased to 188.77 kPa and the LOI value reached 24.9%. In a vertical burning test and a cone calorimeter test, the joint use of APP and DEEP endowed RPUFs with a V-0 rating and they attained a THR value of 23.43 MJ/m^2^. Moreover, the addition of APP improved the acoustic absorption performance of the foam, verified by acoustic absorption measurements. Considering potential applications, the formulation containing 15 php APP and 5 php DEEP could be used in the preparation of a new flame-retardant acoustic absorption rigid polyurethane foam.

## 1. Introduction

Since polyurethane foam was first synthesized in the 1940s, appreciable attention has been paid to them for their wide range of uses in different areas. Common usages such as in tubes, hose products, wall coverings and seals are derived from their ease of processing [1]. Within the family of polyurethane foam, rigid polyurethane foam (RPUF) has attracted extensive interest because of its extremely low thermal conductivity, low permeability and ease of processing. More importantly, its highly developed pore structure gives it potential to be applied in numerous fields. Nowadays, industrial society develops rapidly, accompanied by noise intrusion upon daily life, which may harm people’s mental and physical health. Perplexing and harmful noise arouses interest in developing noise mitigation strategies, and one strategy is to exploit novel and effective acoustic absorption materials. Hence, RPUF has emerged as an interesting candidate, owing to its porous structure meeting the demand of acoustic absorption.

As they propagate through cellular structure, acoustic waves are dissipated. Two mechanisms explain this process: visco-inertial and thermal damping and viscoelastic frame damping [2,3]. In visco-inertial and thermal damping, acoustic pressure in porous media vanishes mainly by viscous friction on cell walls or struts and thermal conduction on solid–fluid interfaces [4,5]. For polymeric foams, visco-inertial and thermal damping dominates the overall acoustic absorption behavior rather than viscoelastic frame damping [6]. Efforts have been devoted to improving the acoustic absorbing properties of RPUF. So far, cell parameters such as size and openness are verified to be decisive factors influencing acoustic damping performance [6,7]. Some researchers have proposed the tuning of raw materials to regulate cell parameters [8,9]. Approaches such as adding various fillers [10,11,12,13,14,15] and composing RPUFs with other materials [16,17] are implemented, as well. The instructive ideology of these works is to increase cells’ structural complexity in order to preserve acoustic waves inside the foams, generating more dissipation.

Nonetheless, the ignitability of RPUF limits its application in building fields [18]; thus, it is worth devoting efforts to promote its flame retardancy. Based on extensive research and practice, phosphorus is thought to be a crucial element exhibiting flame retardancy [19]. Reactive flame retardants containing phosphorus [20,21,22,23,24,25,26] have attracted much attention, yet their applications are limited since phosphorus is expensive and difficult to process. On the contrary, additive flame retardants are widely used for their versatility and availability. Among them, ammonium polyphosphate (APP) is competitive for its high efficiency and low cost. The flame-retarding effect of APP mainly manifests in releasing a noncombustible gas product and forming phosphorus-based substances that catalyze the formation of protective char. Therefore, many researchers aim to exploit more possibilities of APP, such as microencapsulation [27,28,29] and surface modification [30,31]. However, in most situations, a single flame retardant may not achieve satisfactory effects. The joint use of various flame retardants is proposed to construct a multiform synergistic system to meet demands [32,33,34,35,36,37,38,39,40,41,42,43]. As a kind of liquid additive flame retardant, diethyl ethylphosphonate (DEEP) can play a part in gas-phase flame retarding. When heated, DEEP releases active radicals such as HPO· and PO· to interdict chain reactions, performing quenching effects. In consideration of operability and practicability, a binary system constructed by APP and DEEP is promising, yet there is little research on such a system.

Although some encouraging progress has been made, developing appropriate RPUFs with suitable flame retardancy and effective acoustic absorption is less successful and still requires further research. In the present work, RPUFs with open-cell structure were prepared and applied for acoustic absorption. Meanwhile, APP and DEEP were introduced to the RPUFs to improve flame retardancy. A flame-retardant RPUF maintaining acoustic absorption performance is put forward in this paper.

## 2. Materials and Methods

### 2.1. Materials

Polymeric diphenylmethane diisocyanate (PAPI) Suprasec 5005 (viscosity at 25 °C = 160–240 mPa·s, N=C=O content = 30.5–32.5%), a product of Huntsman, was purchased from Guangzhou Hongna Chemical Co., Ltd. (Guangzhou, China). Petrochemical polyether polyol 635 (viscosity at 25 °C = 5500 mPa·s and hydroxyl value = 490 ± 15 mgKOH/g) was obtained from Jining Baichuan Chemical Co., Ltd. (Jining, China). Catalyst Dabco 33LV (triethylenediamine, TEDA) and Niax PC-8 (N,N-dimethylcyclohexylamine, DMCHA) were generously provided by Foshan Daoning Chemical Co., Ltd. (Foshan, China) and Shanghai Sanky Chemical Co., Ltd. (Shanghai, China), respectively. Silicon oil surfactant AK-8801 was purchased from Jiangsu Maysta Chemical Co., Ltd. (Nanjing, China), and was employed as a foam stabilizer. Evonik Industries’ product Ortegol-501 was utilized as a cell opener, obtained from Zhangjiagang Sunbow Chemical Co., Ltd. (Zhangjiagang, China). Distilled water was used as a chemical foaming agent. In this research, ammonium polyphosphate (APP) and diethyl ethylphosphonate (DEEP) were used as flame retardants, supplied by Yangzhou Chenhua Chemical (Yangzhou, China) and Shandong Usolf Chemical (Qingdao, China), respectively. As a fine powder, the particle size of APP ranges from 0 to 35 microns. DEEP is a liquid flame retardant and its viscosity is 1.5 mPa·s. All materials were used without further purification.

### 2.2. Preparation of Rigid Polyurethane Foam

In this work, the rigid polyurethane foam was prepared by a one-pot free-rising method. The polyol, catalyst, foaming agent, cell-opening agent, foam stabilizer, surfactant and flame retardants were added into a plastic beaker and stirred to a uniform mixture with an IKA electric stirrer at a speed of 1000 r/min for 3 min. Subsequently, PAPI was rapidly poured into the above-mentioned mixture and the reaction mixture underwent vigorous stirring at a speed of 1200 r/min for about 10 s. Upon approaching homogeneity, the mixture was poured into a paper mold. A light brown sticky liquid gradually solidified, along with a crosslinking reaction that was complete within tens of seconds. Meanwhile, carbon dioxide generated from the reaction between PAPI and distilled water caused expansion and formed frothy bubbles. Eventually, rigid polyurethane foam was obtained and transferred into an oven to cure at 65 °C for 36 h. After curation, the foams were cut into suitable sizes for further measurements. Details of the foam formulations are listed in Table 1.

### 2.3. Characterization

#### 2.3.1. Fourier Transform Infrared Spectroscopy

The Fourier transform infrared spectroscopy (FTIR) spectra of the flame retardants and prepared samples were recorded on a Nicolet IS50 (Thermo Fisher Scientific, Waltham, MA, USA) using powder-pressed KBr pellets. FTIR spectra were obtained at room temperature in the range of 4000–400 cm^−1^ wavenumber.

#### 2.3.2. Scanning Electron Microscopy

The morphology of the original samples and char after the vertical burning tests were examined by the Hitachi SU 8220 scanning electron microscope. Samples were immersed in liquid nitrogen to embrittle, and then cut into slices. All samples were treated with gold spraying. The accelerating voltage was 15 kV, and the magnification was set at 30 times. The images of foams were analyzed by Image-Pro Plus 6.0 in order to obtain the cell diameter.

#### 2.3.3. Apparent Density Measurement

The apparent density of the samples was measured as specified in ISO 845:2006 (ASTM D1622). Samples were cut into a size of 50 × 50 × 20 mm^3^. The samples were measured and weighed to an accuracy of 0.01 mm and 0.01 g, respectively.

#### 2.3.4. Compressive Strength

The compressive strength test was performed by the AG-IC 50KN Shimadzu universal testing machine according to ISO 844:2004 (ASTM D1621). The compressive strength parallel to the direction of foam rise was investigated. The sample size was 100 × 100 × 50 mm^3^ and the compressive force was applied at a speed of 2 mm/min axially in the perpendicular direction to the square surface. The compressive strength was calculated according to the stress at 10% deformation.

#### 2.3.5. Acoustic Absorption Measurement

The acoustic properties of the foams were evaluated by the acoustic absorption coefficient at the frequency of 125 to 5000 Hz based on ISO 10534-1:1996 (ASTM E1050-2012). The tests were performed on the AWA6290Z Acoustic Absorption Coefficient System, which comprised a standing wave tube, a sound box and a frequency analyzer. The acoustic absorption coefficient is the average value of *α* at 125, 250, 500, 1000, 2000 and 4000 Hz. Foams were tailored in the shape of a cylinder, with diameters of 30 mm and 98 mm for the high and low frequency tests, respectively, and both were 80 mm thick.

#### 2.3.6. Thermogravimetric Analysis

Thermogravimetric analysis was conducted with a simultaneous thermal analyzer, NETZSCH STA449C. The measurement atmosphere was a dry air atmosphere, and the gas flow rate was set at 20 mL/min. The relative mass loss of the samples was recorded from 40 °C to 750 °C at a heating rate of 10 °C/min.

#### 2.3.7. Limited Oxygen Index (LOI)

LOI tests were conducted using an oxygen index instrument based on ISO 4589-2:1996 (ASTM D2863) standard procedure. A specimen with the dimensions of 100 mm × 10 mm × 10 mm was placed in the middle of a glass tube and a mixture of oxygen and nitrogen of known composition passed through. The specimen was ignited at the upper end. The volume fraction of oxygen that permitted steady burning was determined. For each specimen, the LOI test was conducted three times, with an error value of ±0.3%.

#### 2.3.8. Vertical Burning Test

The UL-94 vertical burning test was performed on the CZF-3 instrument (Jiangsu Analysis Instrument Co., Ltd., Xuzhou, China) according to ASTM D3014-04a. The dimensions of the specimen were 200 mm × 15 mm × 15 mm.

#### 2.3.9. Cone Calorimeter Test

The cone calorimeter test was carried out to investigate the combustion behavior of all samples on the instrument of VOUCH (Suzhou Testing Technology Co., Ltd., Suzhou, China) according to ISO 5660-1 (ASTM E1354). The heat flux was 35 kW/m^2^ and the size of the specimen was 100 × 100 × 300 mm^3^.

## 3. Results and Discussion

### 3.1. FTIR Spectra of Samples and Flame Retardants

**Figure 1:**
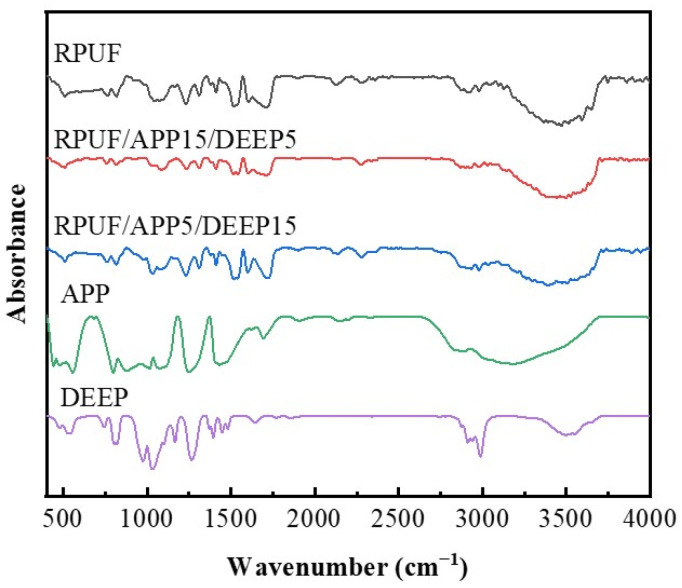
FTIR spectra of RPUF, RPUF/APP15/DEEP5, RPUF/APP5/DEEP15, APP and DEEP.

The FT-IR spectra of prepared foams with APP and DEEP are shown in Figure 1. Due to the existence of the generated N-H and the remaining O-H groups from polyether polyol [44], a band in the spectra of prepared foams between 3000 cm^−1^ and 3500 cm^−1^ can be observed. The peak at 1595 cm^−1^ can be assigned to N-H bending vibration. Peaks at 2978 cm^−1^ represent the CH_2_-CH_2_ stretching vibration [45]. Due to the N=C=O stretching vibration of unreacted isocyanide groups, an absorption band with a peak can be seen at 2270 cm^−1^, mainly caused by the extra amount of PAPI. These excessive isocyanate groups reacted with active hydrogen, and self-polymerization took place to form the polyisocyanate structure, which enhanced the density and hardness of the foams. Peaks at 876 cm^−1^ and 1083 cm^−1^ represent the symmetric and asymmetric stretching vibrations of P-O groups from APP, respectively. Moreover, peaks at 1256 cm^−1^ and 3200 cm^−1^ correspond to the stretching vibration of the P=O and N-H groups from APP, respectively [46]. In the spectrum of DEEP, peaks at 876 cm^−1^ and 1083 cm^−1^ can be assigned to the stretching vibration of P-O groups, and the strong peak at 1256 cm^−1^ corresponds to the stretching vibration of the P=O. These results indicate that RPUF and flame-retardant RPUFs were successfully synthesized.

### 3.2. Effect of Flame Retardants on Cell Morphology, Density and Compressive Strength of Rigid Polyurethane Foam

**Figure 2:**
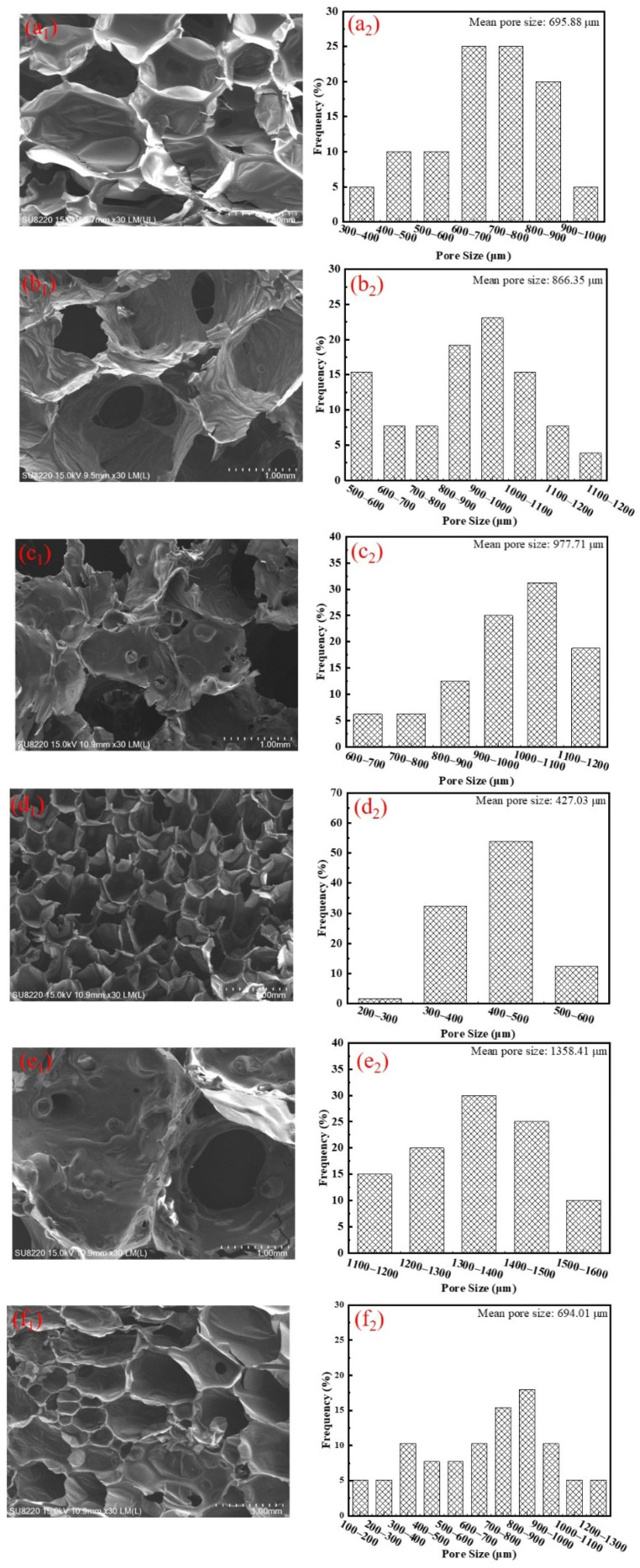
SEM images and pore size distribution of prepared foams. (**a_1_**,**a_2_**) RPUF, (**b_1_**,**b_2_**) O-RPUF, (**c_1_**,**c_2_**) RPUF/APP20, (**d_1_**,**d_2_**) RPUF/DEEP20, (**e_1_**,**e_2_**) RPUF/APP15/DEEP5, (**f_1_**,**f_2_**) RPUF/APP5/DEEP15.

Cell morphology and pore size have a significant influence on the acoustic absorption ability and mechanical properties of the samples [2]. SEM images and corresponding cell size distribution graphs of the prepared foams are presented in Figure 2, all of which display the typical cellular structure of common RPUFs. As displayed in Figure 2a_1_, a mainly closed-cell structure with a diameter of about 0.5–1 mm is in the shape of a polyhedron with smooth cell walls. The films separating contiguous cells are intact. The cell morphology of O-RPUF, which was fabricated by introducing a cell-opening agent, is also depicted in the figure. Apertures on the cell walls derived from the impact of the cell-opening agent are clearly seen, connecting individual cells to form interconnection channels inside the foam matrix. Here, the influence of the cell-opening agent on cell structure is certified by the SEM image, in accordance with previous research [6,47]. Moreover, these open windows between cells offered chances for CO_2_ to transfer before curing, therefore endowing O-RPUF with a mean pore size of 866.35 μm, 24.5% larger than the pristine RPUF. For foams containing APP, small particles were embedded in the cell walls, and formative knots are discernible in Figure 2c_1_,f_1_. The relatively regular shape of cell morphology and flatness of the cell walls were apparently damaged. These alterations are likely due to the fact that the APP may affect the process of cell nucleation in the preparation of RPUF [48]. Nevertheless, unlike the results in the literature reporting that the addition of APP could significantly decrease the cell size [37], a decrease in size could be hardly observed here. This might be due to the high dosage of water, which was used as a chemical blowing agent, covering the influence brought by APP. In contrast, the addition of DEEP obviously changed cell diameter and size distribution, resulting in more homogeneous and less anisotropic foams, as confirmed in Figure 2d_1_. In the synthesis of RPUFs, DEEP not only served as a flame retardant but also as an efficient viscosity reducer [33]. Decreasing the viscosity of preliminary material helped improve the tractility of a polymer film generated by polymerization, prohibiting the coalescence of bubbles and promoting the formation of small, dense cells. To summarize, the addition of 20 php APP had negative effects on cell morphology, while 20 php DEEP had positive effects. Comparing RPUF/APP15/DEEP5 and RPUF/APP5/DEEP15, it is worth noting that the cell structure of the latter appeared to be heterogeneous, with uneven cell sizes, which could be explained by the high viscosity of the reacting mixture with a high dosage of APP [49].

**Figure 3:**
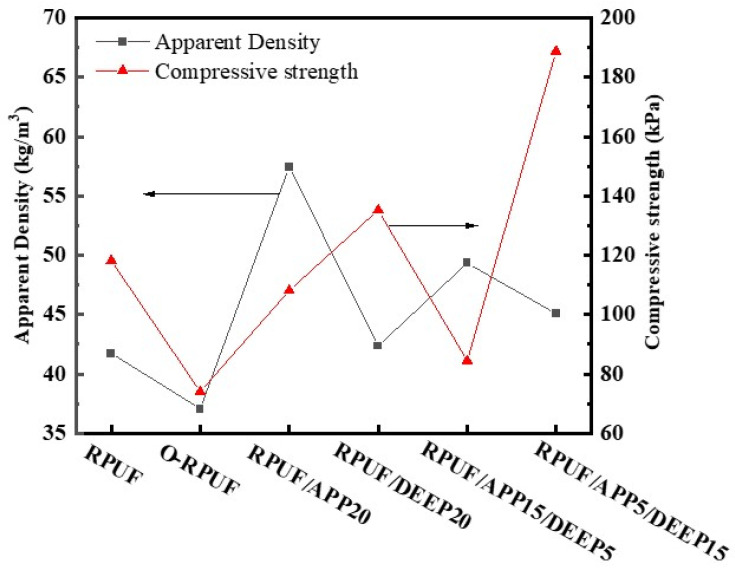
Relationship between apparent density and compression strength of various RPUFs.

Various ingredients significantly influence the density and compressive strength of RPUFs [50]. Apparent density and compressive strength tests of prepared foams were carried out and their results are presented in Figure 3. Table 2 provides comparison of numerical results corresponding to the samples. In contrast to the density of pristine RPUF, the density of O-RPUF dropped to 37.08 kg/m^3^, which could be ascribed to the effect of the cell-opening agent. In the process of foam synthesis, the pore-opening agent caused films between cells to break, forming the open-cell structure, which was beneficial for the gas trapped in the bubbles to spill out. When the curation of foam is not complete, the gas helps bubbles grow larger, leaving the volume with room to grow and resulting in the decrease in the foam’s density. It is worth noting that the density of foams essentially appeared incremental after the addition of flame retardants. Notably, the addition of APP caused a substantial increase in density, attaining 57.45 kg/m^3^, while 20 php DEEP aroused a slight increase to 42.36 kg/m^3^. Moreover, when APP and DEEP were both added to fabricate RPUF/APP/DEEP composites, it could be concluded that higher content of APP led to higher density of the foams.

The compressive strength of RPUF signifies the ability to resist the deformation and collapse of cell units. As expected, the compressive strength of O-RPUF dropped sharply to 74.03 kPa, decreasing by 37.4% compared to pristine RPUF. Some researchers claim that the strength results of polyurethane foam depend on the closed-cell content [51]. Evidently, in the present work, an open-cell structure was distinguished for O-RPUF from the corresponding SEM images. The apertures on the cell walls weakened the foam’s ability to resist compression force, illustrating that the rise in the open-cell rate inevitably caused the decline in compressive strength, coinciding with the results reported by Wang et al. [47]. In comparison with O-RPUF, the compressive strength of RPUF/APP20 reached 108.14 kPa, likely owing to the reinforcement of inorganic fillers [52]. APP is a kind of superfine powder and can be filled into some polymers to enhance flame retardancy and strengthen mechanical properties at the same time. However, some studies point out that with certain additive amounts, stress concentration can occur, since an uneven mixing of fillers induces an internal defect of the foam [27,53,54]. When exceeding a certain additive value, APP particles begin to agglomerate and form relatively large particles, and the asymmetrical dispersion could destroy the continuity of polymer films between cells, causing stress concentration at these sites [34]. Moreover, compressive strength of 135.26 kPa was attained by RPUF/DEEP20, indicating that the addition of DEEP seemed to be desirable to improve the mechanical properties of RPUF. DEEP advanced the regularity of cell structure, consequently promoting stress dispersion when compressed. Compared with O-RPUF, RPUF/APP5/DEEP15 possessed higher compressive strength since the addition of DEEP improved the dispersion state of APP in the matrix. However, RPUF/APP15/DEEP5 had lower compressive strength, which could be attributed to, as mentioned above, the agglomeration of APP particles destroying the continuity of polymer films between cells.

### 3.3. Acoustic Absorption of Rigid Polyurethane Foam

**Figure 4:**
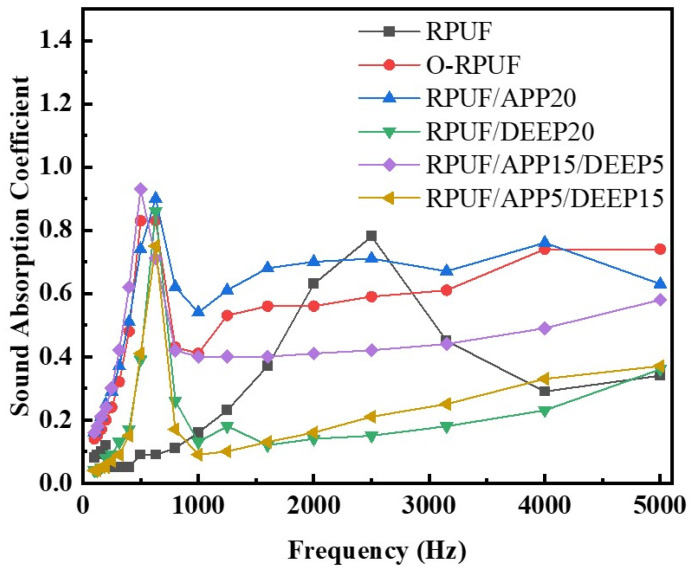
Acoustic absorption coefficient–frequency curves ranging from 125 to 5000 Hz of various RPUFs.

The standing wave ratio method is often implemented in labs to characterize the acoustic absorption performance of materials. The acoustic absorption coefficient–frequency curves ranging from 125 to 5000 Hz of various samples are shown in Figure 4. Additionally, the average absorption coefficient was calculated to evaluate the overall acoustic absorbing performance for the measured frequency, as listed in Table 3. Pristine RPUF exhibited poor acoustic absorbing performance, with an average absorption coefficient of 0.232 due to the dominant closed-cell structure, which could not provide incident aisles to propagate acoustic waves [6]. Thus, we concluded that RPUF was not absorption-sensitive under the given situation. Other samples’ curves showed discernible peak values over the range of 400–700 Hz, suggesting that they obtained optimal acoustic absorption efficiency at medium frequency, likely owing to the resonance effect [2]. O-RPUF absorbed sound more effectively than pristine RPUF, with an average acoustic absorption coefficient of 0.488. In addition, O-RPUF showed remarkable acoustic performance, with its coefficient being higher than 0.5 at frequencies from 1000 Hz to 5000 Hz. As shown in Figure 2a_2_,b_2_, the pore size of O-RPUF was mainly 800–1100 μm, and the mean pore size was 24.49% larger than pure RPUF. These results prove that the promotion of acoustic absorption was achieved by an open-cell structure and larger pore size. Open-cell structure, connecting the inner part of the foam to the atmosphere, has been demonstrated as an indispensable structure for acoustic absorption materials [6,7,9]. Moreover, interconnected cells with larger diameters in the foam matrix crucially affect absorption performance [55]. Acoustic waves are able to spread into the foam matrix through these aisles. While spreading in cavities inside the foam, friction between air molecules and cell walls arouses acoustic energy, which converts into heat and is then dissipated. A larger cell size provides more room for the sound waves to enter the foam, and thus, the energy of sound can be dissipated more easily. Consequently, an open-cell structure and larger cell size endowed O-RPUF with a better ability to absorb sound than RPUF.

Moreover, favorable results were obtained for RPUF/APP20. The average coefficient climbed up to 0.535 and a maximum value of 0.9 was gained at 630 Hz. The acoustic absorption coefficient exceeded 0.6 over frequencies higher than 1500 Hz. The addition of APP facilitated considerable acoustic absorption of O-RPUF. One possible explanation is that flow resistivity and pore tortuosity, which have crucial influence on the acoustic absorption of materials, were improved [15]. As depicted in Figure 2c_1_, APP particles were enclosed in the cell walls, forming coarse surfaces that increased the contact area. Acoustic waves propagated through a more tortuous path, indicating that waves were more likely to be interrupted and that dissipation occurred in the foam matrix. In addition, the improvement of acoustic absorption of the RPUF filled with solid particles could be attributed to the increased energy dissipation as heat through hysteresis [56]. According to Figure 2c_2_, the pore size distribution of RPUF/APP20 was mainly in the range of 900–1100 μm, similar to that of O-RPUF. The appropriate pore size distribution of RPUF/APP20 gave the foam similar conditions to O-RPUF for sound absorbing, indicating the optimal cell size distribution. However, the average acoustic absorption coefficient of RPUF/DEEP20 was only 0.17. As depicted in Figure 2d_1_,d_2_, incorporating DEEP could enhance the regularity of the foam’s morphology and reduce the overall pore size. Hence, sound waves did not penetrate deep into the foam matrix and their propagation was blocked, similar to the situation of RPUF.

While using APP and DEEP simultaneously, an average absorption coefficient of 0.452 and a maximum value of 0.93 at 500 Hz were obtained for RPUF/APP15/DEEP5, much higher than those of RPUF/APP5/DEEP15, further indicating that APP did enhance the acoustic absorption of RPUF. In general, since APP and DEEP displayed different effects on cell structure and pore size, upon which the acoustic properties of RPUF highly depend [57], they showed opposite effects on the acoustic absorption performance of RPUF. When APP and DEEP were added to RPUF simultaneously, APP led to irregular open cells while DEEP led to small, closed cells. Some researchers pointed out that solid fillers favored forming an open-cell structure, resulting in better acoustic absorption performance [58,59]. A more open-cell structure was gained with higher APP content and the embedment of APP increased the stiffness of the cell walls, hence increasing the scattering or reflection of sound waves in cavities [14]. On the contrary, higher content of DEEP led to a closed-cell structure that blocked sound waves from propagating into the foam and exhibited poor acoustic absorption.

### 3.4. Thermal Stability of Rigid Polyurethane Foam

**Figure 5:**
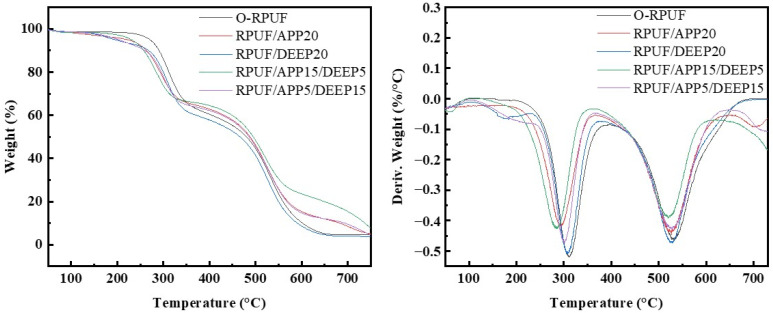
Thermogravimetric (TGA) and differential thermogravimetric (DTG) curves of different samples.

Thermogravimetric analysis under a dry air atmosphere was carried out to characterize the thermal stability of the foams for further probe into the decomposition process. Figure 5 reveals the TG and DTG curves of various samples, and corresponding parameters are listed in Table 4. All specimens showed their main pyrolysis stage between 250 and 650 °C, and distinct peaks were discerned at temperatures ranging around 250–550 °C. As demonstrated, O-RPUF emerged with a two-step degrading process, coinciding with results in the literature [35,60]. Inchoate mass loss was ascribed to the evaporation of remnant moisture or other unreacted molecules. As the temperature increased, thermal degradation of the foam deepened. The pyrolysis procedure can be summarized as followed: the preliminary decomposition of RPUF matrix took place at around 290 °C with the breakage of main chemical bonds and the decomposition of small molecules. Specifically, in the range of 200–450 °C, urethane bonds depolymerized into isocyanate and polyol. Then, as temperature gradually came to 311.8 °C, the mass loss rate reached the first peak value. In the range of 450–600 °C, macromolecule segments and main crosslinking networks were mostly broken with the escape of small gas molecules such as HCN, NO, CO, etc. [18,61]. At this stage, aromatic compounds decomposed and carbonized even further. Finally, residual mass remained only 4.52%.

In the case of RPUF/APP20, T_5%_ saw a general decline to 222.0 °C from 270.9 °C. The reduction in the initial decomposition temperature was mainly caused by the elimination of NH_3_ and H_2_O during the thermal degradation process of APP. Furthermore, APP substantially brought the first maximum mass loss rate peak forward to lower temperatures, as reflected by Tmax1. In general, the incorporation of APP lowered the initial decomposition temperature and maximum mass loss rate temperature of the first stage, coinciding with a previous report [35]. The second stage began at approximately 350 °C, which could be attributed to the formation of polyphosphoric acid. The formation of char catalyzed by polyphosphoric acid became a compact layer and inhibited the mass loss [30]. Different pyrolysis behaviors were found in RPUF/DEEP20. As listed in Table 4, T5% of RPUF/DEEP20 decreased even further below 200 °C because the evaporation of DEEP before the boiling point led to a rapid loss, in contrast with O-RPUF [62,63]. DTG curves indicated a different trend compared to the results of RPUF/APP20, sharing comparable shapes with those of O-RPUF. DTG curves disclosed that the mass loss rate of RPUF/DEEP20 observably decreased in the first and second decomposition stages.

As APP and DEEP were simultaneously employed in RPUF, a joint effect was distinctly discerned. RPUF/APP15/DEEP5 possessed higher residual mass than that of RPUF/APP5/DEEP15. Moreover, DTG curves demonstrated that flame-retardant RPUFs reached their maximum mass loss rate at a lower temperature. The joint effect of APP and DEEP on the pyrolysis behavior of RPUFs can be concluded as follows. On one hand, both decomposed at lower temperatures, indicating that the pyrolysis of RPUF began at an early stage. As temperature rose, phosphorus-containing substances such as phosphoric acid were generated from the decomposition of APP and these substances catalyzed the carbonization of RPUFs [33]. An intumescent carbon layer was then formed and covered the surface, protecting the foam matrix from the intrusion of heat flux for further pyrolysis.

### 3.5. Flame Retardancy of Rigid Polyurethane Foam

#### 3.5.1. Morphology of Residual Char

**Figure 6:**
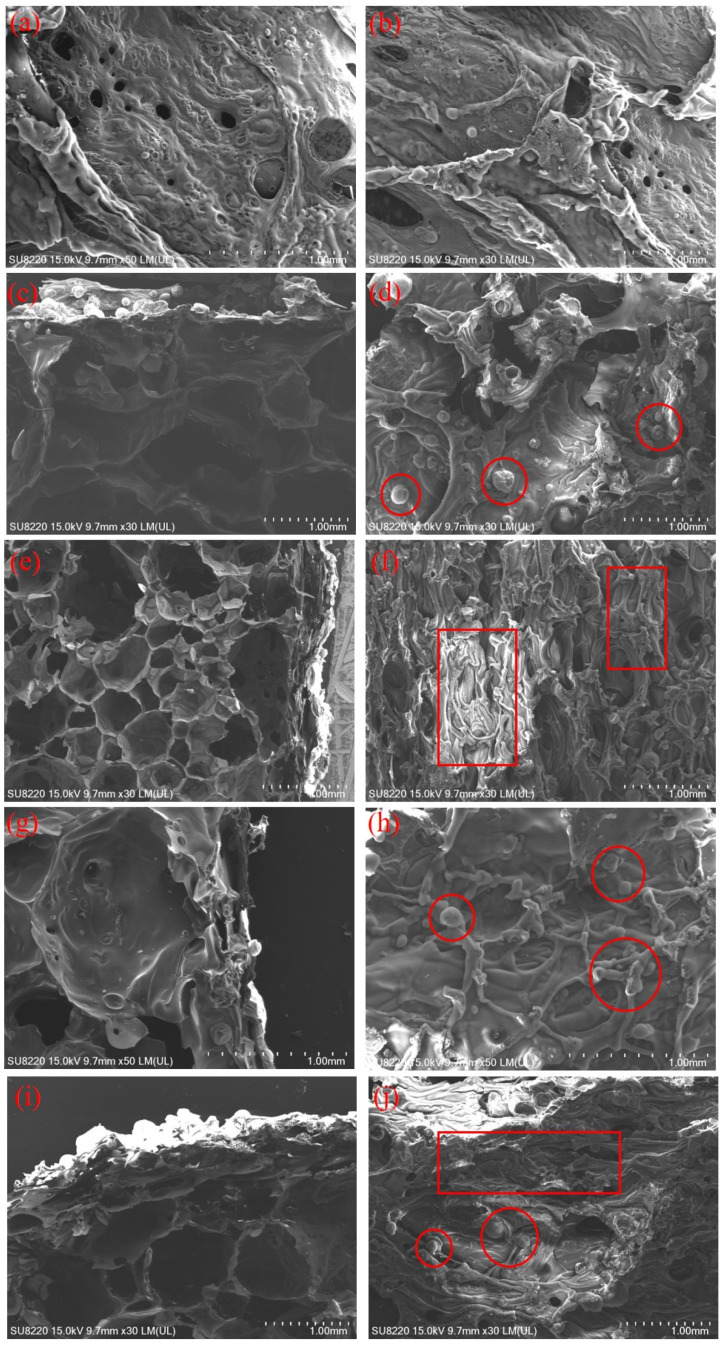
SEM images of char residue after vertical burning tests of RPUF loading different kinds and contents of flame retardants. (**a**,**b**) Surface of burnt O-RPUF; (**c**,**d**) cross section and surface of burnt RPUF/APP20; (**e**,**f**) cross section and surface of burnt RPUF/DEEP20; (**g**,**h**) cross section and surface of burnt RPUF/APP15/DEEP15; (**i**,**j**) cross section and surface of burnt RPUF/APP5/DEEP15. Small particles are framed using circles and parts of the corrugated surface are framed using rectangles.

To further clarify the flame retarding performance, morphologies of the char residues for different samples were observed. Figure 6 shows SEM images of char residue of chosen samples’ cross sections and surfaces after vertical burning tests. The char residue morphology of O-RPUF is shown in Figure 6a,b. It can be clearly seen that numerous holes were formed during combustion, and the original smooth cell walls collapsed and shrank. Some crater-like structures and large holes appeared, causing mass change between the matrix and the atmosphere. Hence, mass and heat transferred through these aisles to maintain continuous combustion. From Figure 6c–j, the images of the cross sections depict explicit boundaries of the pyrolysis front, and the completely undamaged foam structure was preserved. In contrast, O-RPUF experienced complete combustion, and the foam structure was fully destroyed. This could be interpreted as a result of excellent thermal insulation of the foam and blocking effects brought by flame retardants [64]. Figure 6c,d show the char residue morphology of RPUF/APP20. A few particles (framed out using circle) were found, and the continuity and compactness were promoted. The compact char layer prevented the spreading of external oxygen and combustible gas. Different morphology was found for RPUF/DEEP20. As displayed in Figure 6e,f, the char layer of RPUF/DEEP20 was thinner in contrast with RPUF/APP20. Holes below the char layer were observed and the exterior surface was more corrugated (framed out using rectangle), confirming that the char layer did not close the mass change channel. Meanwhile, DEEP showed flame retardancy in a different way, acting mainly in the gaseous phase. Instead of staying in a condensed phase like APP, DEEP volatilizes at lower temperatures and decomposes into free radicals, blocking the chain reaction of combustion [33]. Concerning the char residue morphology of RPUF/APP5/DEEP15 and RPUF/APP15/DEEP5, they were similar and both characteristics were found. Small particles and corrugated surfaces appeared simultaneously. With higher APP content, more particles were observed. In contrast, with higher DEEP content, the surface of the char was more corrugated.

#### 3.5.2. LOI, Vertical Burning and Cone Calorimeter Tests Results

Aiming at elucidating the influence of different flame retardants on the foams, vertical burning tests, LOI tests and cone calorimeter tests were conducted. The LOI value represents the minimum concentration of oxygen in a flowing mixture of oxygen and nitrogen that supports the steady burning of given materials, which has a certain reference for practical application. Polyurethane foam without flame retardants is flammable and its LOI value may reach only 18–19% [65]. The results of different specimens for LOI tests are listed in Table 5. O-RPUF had the lowest LOI value of 18.6% among all specimens, suggesting that O-RPUF could be easily ignited and maintain continuous combustion in a normal atmosphere. By using 20 php APP separately, the LOI value was elevated from 18.6% to 22.7%, indicating a reduction in flammability. Regarding the RPUFs consisting of APP and DEEP, the binary flame-retarding system granted RPUFs similar LOI values, and both of them exhibited better performances than RPUF containing APP alone. When 5 php APP and 15 php DEEP were incorporated into RPUF, the LOI value of the foam reached 24.9%. This could be explained by the joint use of APP and DEEP, which produced a barrier layer to inhibit the matrix from decomposition [42]. Hence, the adiabatic carbon layer covered the surface and prevented the mass and heat transfer.

Along with the LOI test, the vertical burning test was also used to assess the flame retardancy of materials. The results of vertical burning tests are summarized in Table 5. None of the specimens exhibited droplets in the experiments. O-RPUF burned violently, with the release of thick, black smoke once ignited by the source fire, maintaining a residual mass fraction of 31.67%. Fierce combustion, coupled with long after-flame time up to 45 s, rendered O-RPUF a failure of classification. Likewise, failing to classify also appeared for RPUF/APP20 for its long after-flame time. Nevertheless, the introduction of APP dramatically decreased after-flame time by 66.7% and residual mass fraction increased to 80.82%. For RPUF/DEEP20, after-flame time was just 1.5 s, graded as V-0, and 86.58% residual mass was obtained. RPUF containing both APP and DEEP had similar after-flame time and residual mass, being classified as V-0, as well. It was worth mentioning that the samples sparked and made a crackling sound for a short time when moved away from the ignition source, then extinguished suddenly. In other words, quick self-extinguishing results were achieved for the RPUF/APP/DEEP formula. This is strong evidence of the better flame retardancy of RPUF/APP/DEEP.

**Figure 7:**
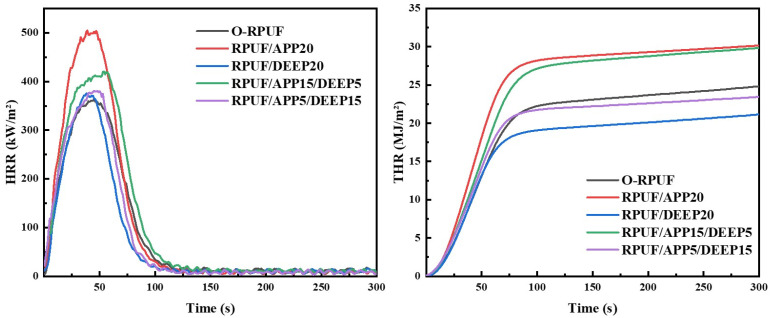
HRR curves and THR curves of O-RPUF, RPUF/APP, RPUF/DEEP and RPUF/APP/DEEP.

Moreover, the combustion behaviors of O-RPUF, RPUF/APP, RPUF/DEEP and RPUF/APP/DEEP were investigated by cone calorimeter tests. As displayed in Figure 7, the total heat release of RPUF/APP20 was the largest among the tested samples, while the total heat release of RPUF/DEEP20 was the smallest. It can also be observed from Figure 7 that the heat release rate curve of RPUF/APP20 was much higher than that of RPUF/DEEP20 at 50–100 s. After increasing the amount of DEEP, the total heat release began to decrease. These results were consistent with the conclusion obtained in the thermogravimetric tests. DEEP evaporated during the heating process, and the free radicals released during the degradation process could effectively prevent the occurrence of a chain reaction for pyrolysis. These two steps fully reduced the heat released by the foam during combustion. As illustrated in Table 6, the total smoke production of RPUF/DEEP20 was the lowest among the samples with flame retardants, which also benefited from the efficiency of DEEP in hindering combustion and oxidation reaction. As a result of complete combustion, O-RPUF had the lowest total smoke generation of all tested samples. Without the intervention of flame retardants, O-RPUF burned more fully, so the output of incomplete oxidized particles decreased, resulting in the decrease in smoke production, and vice versa. Concerning time to ignition, adding one flame retardant alone could not effectually improve the flame retardancy performance. After adding two flame retardants, the combustion time exceeded 7 s, proving the synergistic flame-retardant effect of APP and DEEP.

Based on previous discussion, the synergistic flame retarding effects of APP and DEEP on RPUF are proposed as follows. At relatively low temperatures, the evaporation of DEEP diluted the gas concentration involved in combustion [33,63]. Meanwhile, phosphorous-containing free radicals brought by the decomposition of DEEP captured highly active free radicals produced by the pyrolysis of the foam matrix to stop the chain reaction, exerting a quenching effect. As the temperature increased, APP decomposed into inert NH_3_ gas, diluting the concentration of surface flammable gas. A compact phosphorus-rich char layer was catalytically generated by the polyphosphoric acid, which inhibited the matrix from further pyrolysis [38,66]. Consequently, burning intensity was suppressed and quick self-extinguishing was achieved. The binary flame-retarding system constructed by APP and DEEP demonstrated the ability to perform in gaseous and condensed phases, which can dramatically enhance the RPUF’s flame retardancy.

## 4. Conclusions

Flame-retardant rigid polyurethane foam with an open-cell structure was synthesized and applied for acoustic absorption. Compared with pristine RPUF, an open-cell structure was constructed by adding a cell-opening agent, which was verified by SEM. The addition of APP and DEEP improved the apparent density, compressive strength and flame retardancy of the foam. Regarding flame retardancy, RPUF containing 5 php of APP and 15 php of DEEP achieved an LOI value of 24.9%, gained a V-0 rating in vertical burning tests and obtained a THR value of 23.43 MJ/m^2^. APP facilitated the foam matrix to form an intumescent carbon layer which protected the foam from further decomposition. Meanwhile, DEEP released highly active species to capture free radicals produced by the foam matrix, leading to a rapid self-extinguishing effect. As for acoustic absorption, the open-cell RPUF obtained an average absorption coefficient of 0.488. Moreover, the open-cell RPUF with 20 php APP not only possessed an average absorption coefficient of 0.535 but also gained an absorption coefficient higher than 0.6 at a frequency range of 1500–5000 Hz, showing highly efficient acoustic absorption. The addition of APP can increase coarseness, thus improving friction between acoustic waves and cell walls and resulting in higher efficiency of acoustic energy dissipation. However, incorporating DEEP caused closed cells in O-RPUF, leading to poor acoustic absorption.

## Figures and Tables

**Table 1:** Formulations of rigid polyurethane foams.

Sample	PAPI/php ^a^	Polyether Polyol/ php	33LV/ php	PC-8/ php	AK-8801/ php	Ortegol-501/ php	H_2_O/ php	APP/ php	DEEP/ php
RPUF	105.0	50.0	1.0	1.0	0.5	0	3.0	0	0
O-RPUF	105.0	50.0	1.0	1.0	0.5	2.0	3.0	0	0
RPUF/APP20	105.0	50.0	1.0	1.0	0.5	2.0	3.0	20.0	0
RPUF/DEEP20	105.0	50.0	1.0	1.0	0.5	2.0	3.0	0	20.0
RPUF/APP5/DEEP15	105.0	50.0	1.0	1.0	0.5	2.0	3.0	5.0	15.0
RPUF/APP15/DEEP5	105.0	50.0	1.0	1.0	0.5	2.0	3.0	15.0	5.0

^a^ Units of php means parts per hundred of polyol by weight.

**Table 2:** Values of apparent density and compressive strength tests.

Sample	Apparent Density/(kg/m^3^)	Compressive Strength/kPa
RPUF	41.74	118.21
O-RPUF	37.08	74.03
RPUF/APP20	57.45	108.14
RPUF/DEEP20	42.36	135.26
RPUF/APP15/DEEP5	49.35	84.47
RPUF/APP5/DEEP15	45.08	188.77

**Table 3:** Average and maximum acoustic absorption coefficient of various RPUFs.

Sample	Acoustic Absorption Coefficient		Maximum Frequency (Hz)
	Average	Maximum	
RPUF	0.232	0.46	4000
O-RPUF	0.488	0.83	500
RPUF/APP20	0.535	0.90	630
RPUF/DEEP20	0.170	0.86	630
RPUF/APP15/DEEP5	0.452	0.93	500
RPUF/APP5/DEEP15	0.18	0.75	630

**Table 4:** Thermal degradation parameters of the foams.

Sample	T_5%_/°C	T_max_/°C		Residual Mass/%
		T_max1_/°C	T_max2_/°C	
O-RPUF	270.9	311.8	532.8	4.52
RPUF/APP20	222.0	295.6	527.8	5.11
RPUF/DEEP20	195.4	309.5	526.3	3.80
RPUF/APP15/DEEP5	230.8	285.5	523.2	7.64
RPUF/APP5/DEEP15	202.4	299.7	528.1	4.61

T_5%_ refers to the temperature at 5.0% weight loss of the samples, representing the initial degradation temperature; T_max_ refers to the temperature at maximum rate degradation while the number refers to the maximum rate degradation temperature of different peaks.

**Table 5:** Results of LOI tests and vertical burning tests.

Sample	LOI/%	*t*/s	V-Rating	Dripping	Residual Mass/%
O-RPUF	18.6	45.0	-	No	31.67
RPUF/APP20	22.7	15.0	-	No	80.82
RPUF/DEEP20	24.4	1.5	V-0	No	86.58
RPUF/APP15/DEEP5	24.3	3.0	V-0	No	84.71
RPUF/APP5/DEEP15	24.9	3	V-0	No	83.33

*t*, average after-flame time after the first and second flame impingements; V-rating, rating of vertical burning test according to the grading standard.

**Table 6:** Data of RPUFs from cone calorimeter tests.

Sample	TTI/s	pHRR/kW·m^−2^	THR/MJ·m^−2^	TSP/m^2^
O-RPUF	4	364.37	24.82	3.85
RPUF/APP20	5	504.60	30.17	7.05
RPUF/DEEP20	4	375.29	21.15	5.50
RPUF/APP15/DEEP5	8	420.39	29.84	7.36
RPUF/APP5/DEEP15	7	380.91	23.43	6.95

TTI, time to ignition; pHRR, peak heat release rate; THR, total heat release; TSP, total smoke production.

## Data Availability

The data presented in this study are available on request from the corresponding author.
